# Supplementary material for: Recurrent evolutionary switches of mitochondrial cytochrome c maturation systems in Archaeplastida
Source: Nat Commun. 2024 Feb 20;15:1548. doi: 10.1038/s41467-024-45813-y (PMC10879542; doi:10.1038/s41467-024-45813-y)
Supplement: Supplementary file 1 — Supplementary Information [file 41467_2024_45813_MOESM1_ESM.pdf]

Recurrent evolutionary switches of mitochondrial cytochrome *c* maturation systems in Archaeplastida

Li *et al.*

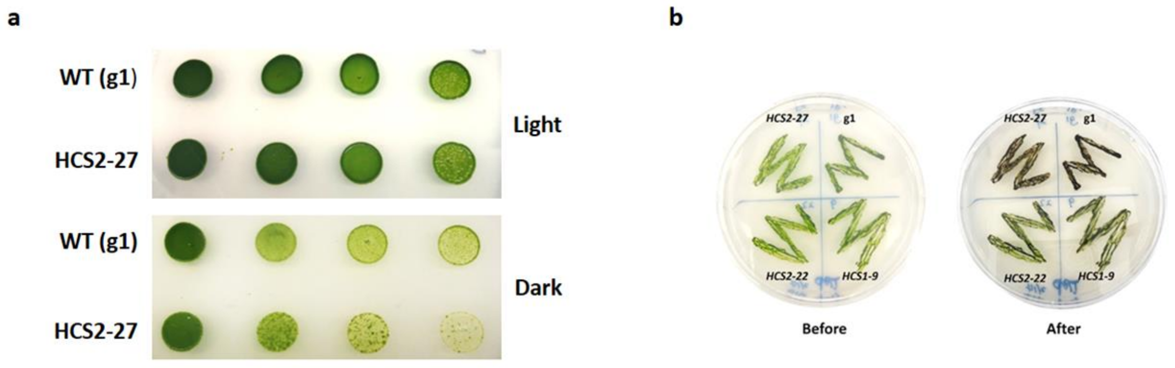

Supplementary Fig. 1. Spot test assay and TTC staining of *Chlamydomonas* HCS mutants. a, The in-frame HCS2-27 mutant exhibits growth deficiency in the dark. **b**, Compared to the WT g1 strain and HCS2-27, knock-out mutants HCS1-9 and HCS2-22 remain green after TTC staining, indicating a disruption of their mitochondrial ETC functionality.

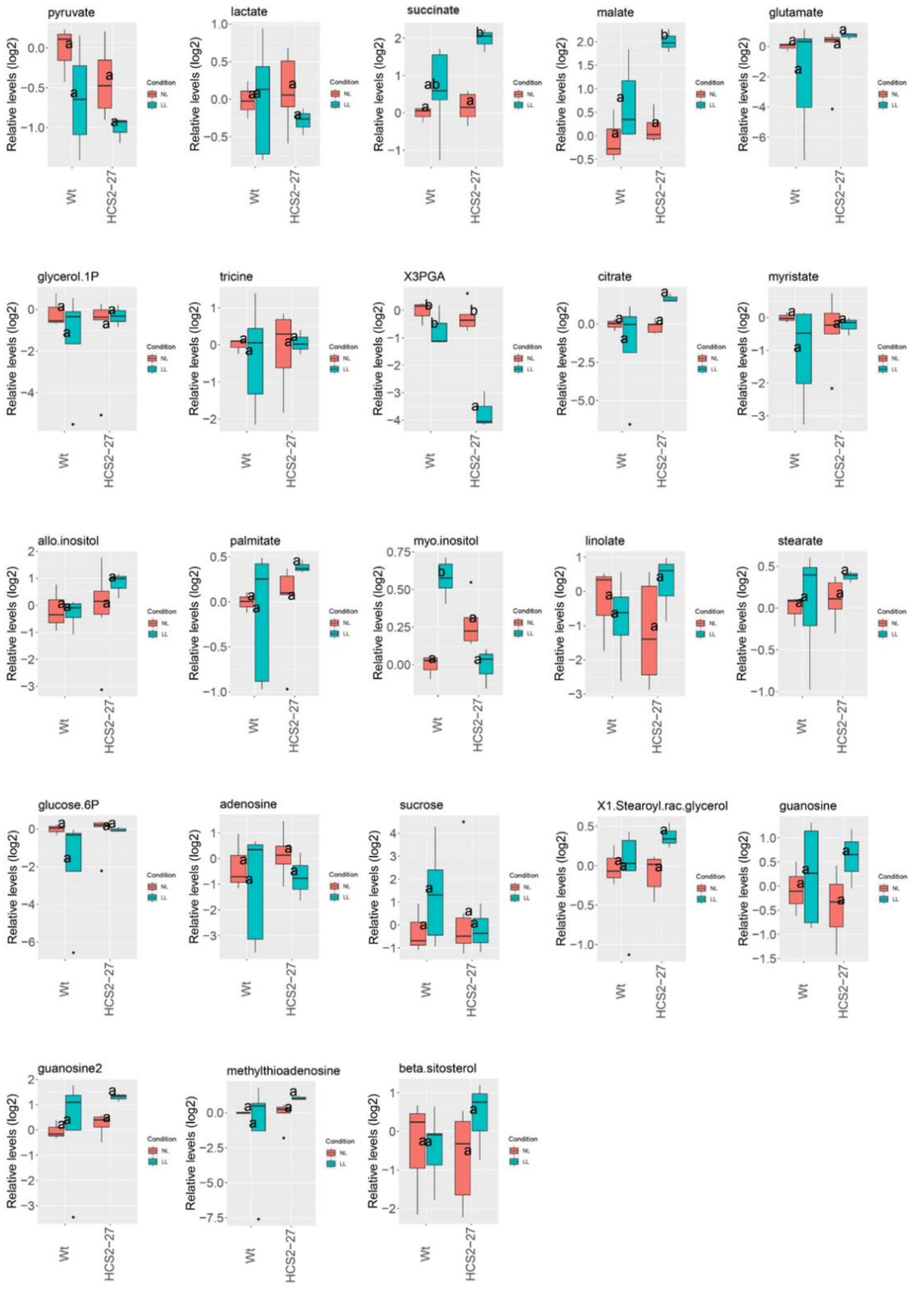

Supplementary Fig. 2. Metabolomic analysis of *Chlamydomonas* HCS knock-down mutant (HCS2-27) and the wild type (Wt) grown under normal light (NL) and low light (LL) conditions. The relative metabolite levels were normalized by the mean of those in Wt/NL conditions and log2 transformed. The boxplots show the minimum, first quartile, median, third quartile, and maximum relative levels (n = 3, 6, 4, 3 for each sample). Letters indicate significant differences between mean values (one-way ANOVA: Tukey's multiple comparisons test, $P < 0.05$), and groups with the same letters are not significantly different. Source data are provided as a Source Data file.

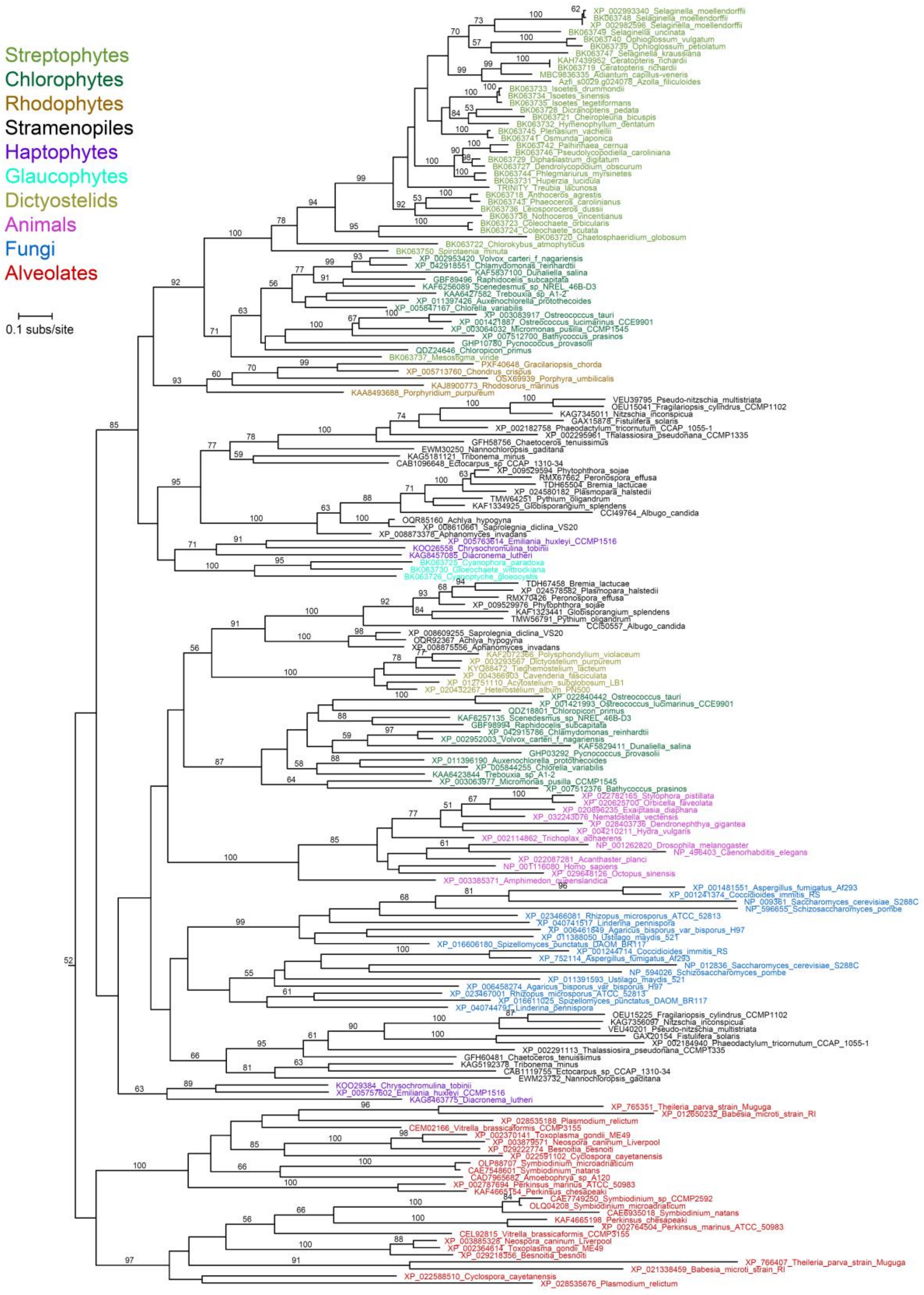

Supplementary Fig. 3. Fully labeled ML tree corresponding to Figure 7A. Bootstrap values <50% not shown. Tree was rooted by midpoint rooting.

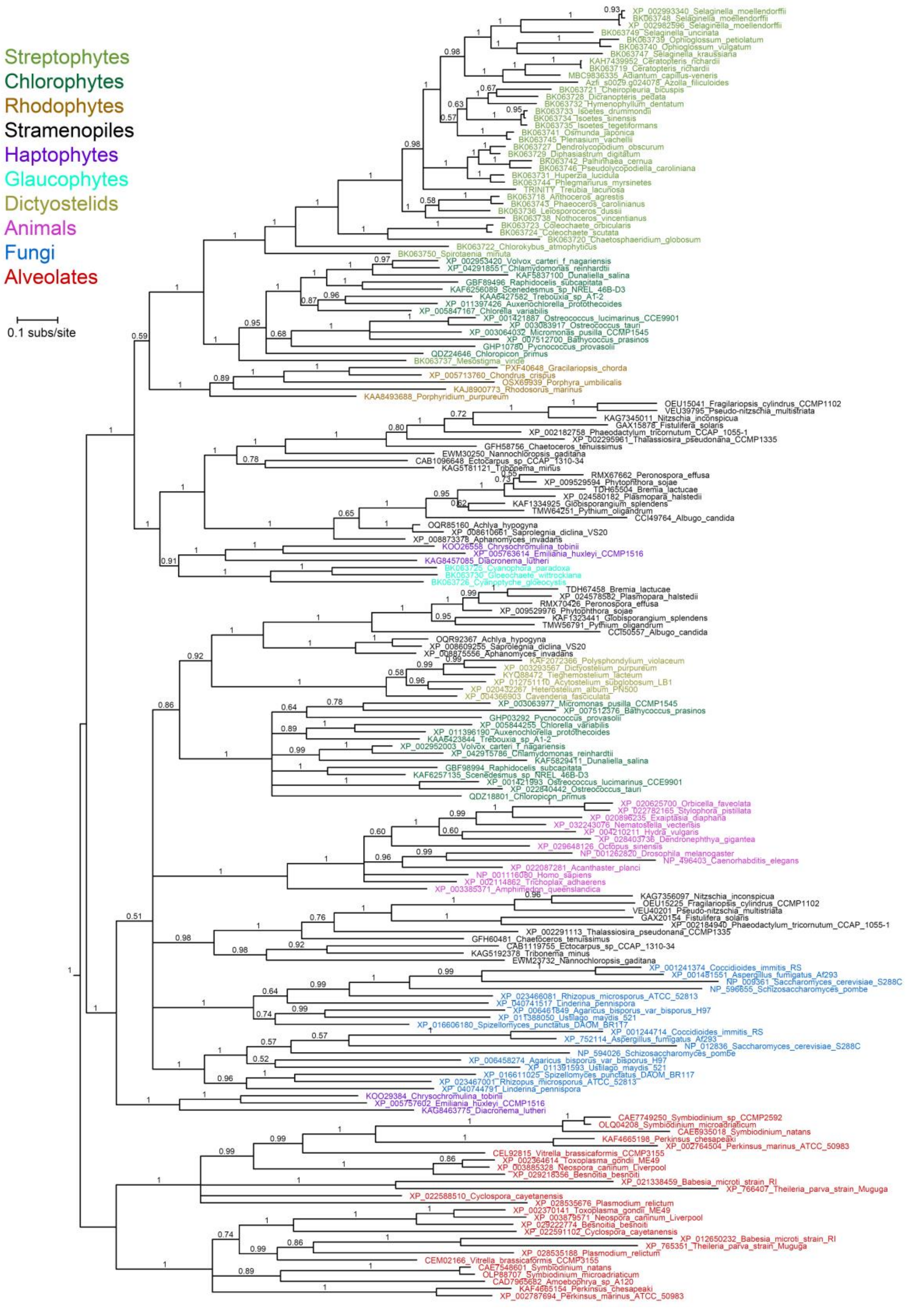

Supplementary Fig. 4. Fully labeled Bayesian tree corresponding to Figure 7A. Posterior probabilities <0.5 not shown. Tree was rooted by midpoint rooting.

Supplementary Table 1. Sequence features and subcellular localization prediction of *C. reinhardtii* HCS.

Gene name	Phytozome ID	Genbank ID	CDS (bp)	5'UTR (bp)	Exon	Intron	Subcellular localization	
							PredAlgo	WoLF PSORT
CreHCS1	Cre12.g525700	5722497	879	359	5	4	M	M
CreHCS2	Cre16.g690050	5724796	960	0	6	5	M	M

CDS, coding sequencing; UTR, untranslated region; M, mitochondrion; PredAlgo (http://lobosphaera.ibpc.fr/predalgo) and WoLF PSORT (https://wolfpsort.hgc.jp/).

Supplementary Table 2. Accession numbers for query sequences used for blast searches.

Species	System I proteins		System III proteins	
Land plants				
Arabidopsis thaliana	CCMA	NP_176516	-	-
	CCMB	YP_009472099	-	-
	CCMC	YP_009472125	-	-
	CCME	NP_190747	-	-
	$CCMF_{C}$	YP_009472113	-	-
	$CCMF_{N1}$	YP_009472126	-	-
	$CCMF_{N2}$	YP_009472124	-	-
	CCMH	NP_563966	-	-
Selaginella moellendorffii	-	-	HCCS	XP_002982596
Green algae				
Chlamydomonas reinhardtii	-	-	HCS1	XP_042918551
	-	-	HCS2	XP_042915786
Klebsormidium nitens	CCMA	GAQ83552	-	-
	CCMB	GAQ93830	-	-
	CCMC	GAQ93831	-	-
	CCME	GAQ86245	-	-
	CCMF	GAQ93806	-	-
	CCMH	GAQ81871	-	-
Red algae				
Cyanidioschyzon merolae	CCMA	BAA36528	-	-
	CCMB	BAA36529	-	-
	CCMC	BAA36544	-	-
	CCME	XP_005536347	-	-
	CCMF	BAA36527	-	-
	CCMH	XP_005534965	-	-
Chondrus crispus	-	-	HCCS	XP_005713760
