# Supplementary material for: Recurrent evolutionary switches of mitochondrial cytochrome c maturation systems in Archaeplastida
Source: Nat Commun. 2024 Feb 20;15:1548. doi: 10.1038/s41467-024-45813-y (PMC10879542; doi:10.1038/s41467-024-45813-y)
Supplement: Supplementary file 3 — Description of Additional Supplementary Files [file 41467_2024_45813_MOESM3_ESM.pdf]

Description of Additional Supplementary Files

Supplementary Data 1: Metabolomics information

Supplementary Data 2: Accession numbers for additional sequencing data used

Supplementary Data 3: Information for transgenic work
