# Supplementary material for: Recurrent evolutionary switches of mitochondrial cytochrome c maturation systems in Archaeplastida
Source: Nat Commun. 2024 Feb 20;15:1548. doi: 10.1038/s41467-024-45813-y (PMC10879542; doi:10.1038/s41467-024-45813-y)
Supplement: Supplementary file 7 — Reporting Summary [file 41467_2024_45813_MOESM7_ESM.pdf]

nature portfolio

Corresponding author(s): JP Mower

Last updated by author(s): Jan 31, 2024

Reporting Summary

Nature Portfolio wishes to improve the reproducibility of the work that we publish. This form provides structure for consistency and transparency in reporting. For further information on Nature Portfolio policies, see our Editorial Policies and the Editorial Policy Checklist.

Statistics

For all statistical analyses, confirm that the following items are present in the figure legend, table legend, main text, or Methods section.

n/a	Confirmed	
☐	☒	The exact sample size (*n*) for each experimental group/condition, given as a discrete number and unit of measurement
☐	☒	A statement on whether measurements were taken from distinct samples or whether the same sample was measured repeatedly
☐	☒	The statistical test(s) used AND whether they are one- or two-sided<br>*Only common tests should be described solely by name; describe more complex techniques in the Methods section.*
☒	☐	A description of all covariates tested
☒	☐	A description of any assumptions or corrections, such as tests of normality and adjustment for multiple comparisons
☐	☒	A full description of the statistical parameters including central tendency (e.g. means) or other basic estimates (e.g. regression coefficient) AND variation (e.g. standard deviation) or associated estimates of uncertainty (e.g. confidence intervals)
☐	☒	For null hypothesis testing, the test statistic (e.g. *F*, *t*, *r*) with confidence intervals, effect sizes, degrees of freedom and *P* value noted<br>*Give P values as exact values whenever suitable.*
☐	☒	For Bayesian analysis, information on the choice of priors and Markov chain Monte Carlo settings
☒	☐	For hierarchical and complex designs, identification of the appropriate level for tests and full reporting of outcomes
☒	☐	Estimates of effect sizes (e.g. Cohen's *d*, Pearson's *r*), indicating how they were calculated

Our web collection on statistics for biologists contains articles on many of the points above.

Software and code

Policy information about availability of computer code

Data collection	blast v2.2.31<br>blastp search (https://blast.ncbi.nlm.nih.gov/Blast.cgi)
Data analysis	CHOPCHOP v2 (https://chopchop.cbu.uib.no)<br>WoLF PSORT (https://wolfpsort.hgc.jp/)<br>PredAlgo (http://lobosphaera.ibpc.fr/predalgo)<br>IQ-TREE v2.2.2.6<br>MAFFT v7.245<br>MrBayes v3.2.7<br>RAxML v8.2.4<br>SPAdes v3.13.0<br>trimAl v1.2<br>Trinity v2.6.6

For manuscripts utilizing custom algorithms or software that are central to the research but not yet described in published literature, software must be made available to editors and reviewers. We strongly encourage code deposition in a community repository (e.g. GitHub). See the Nature Portfolio guidelines for submitting code & software for further information.

Data

Policy information about availability of data

All manuscripts must include a data availability statement. This statement should provide the following information, where applicable:

- Accession codes, unique identifiers, or web links for publicly available datasets
- A description of any restrictions on data availability
- For clinical datasets or third party data, please ensure that the statement adheres to our policy

Sequence data generated in this study have been deposited in GenBank (accessions BK063718–BK063750) and the NCBI SRA (accessions SRR24748178–SRR24748183). Source data are provided with this paper.

Research involving human participants, their data, or biological material

Policy information about studies with human participants or human data. See also policy information about sex, gender (identity/presentation), and sexual orientation and race, ethnicity and racism.

Reporting on sex and gender	Not applicable
Reporting on race, ethnicity, or other socially relevant groupings	Not applicable
Population characteristics	Not applicable
Recruitment	Not applicable
Ethics oversight	Not applicable

Note that full information on the approval of the study protocol must also be provided in the manuscript.

Field-specific reporting

Please select the one below that is the best fit for your research. If you are not sure, read the appropriate sections before making your selection.

☒ Life sciences ☐ Behavioural & social sciences ☐ Ecological, evolutionary & environmental sciences

For a reference copy of the document with all sections, see nature.com/documents/nr-reporting-summary-flat.pdf

Life sciences study design

All studies must disclose on these points even when the disclosure is negative.

Sample size	For Chlamydomonas growth curve measurement, we used 3 biological replicates for each strain.<br>For oxygen consumption measurement, we used the following number of biological replicates for each strain under each condition: WT-Dark N=6; HCS2-27-Dark N=7; HCS2-22-Dark N=3; HCS1-9-Dark N=3; WT+NaCN N=5; HCS2-27+NaCN N=4; HCS2-22+NaCN N=3; HCS1-9+NaCN N=3; WT+SHAM N=5; HCS2-27+SHAM N=3; HCS2-22+SHAM N=3; HCS1-9+SHAM N=3; WT+NaCN+SHAM N=3; HCS2-27+NaCN+SHAM N=3.<br>For metabolite profiling, we used the following number of biological replicates for each strain under each condition: WT-NL N=3; HCS2-NL N=6; WT-LL N=4; HCS2-LL N=3.<br>For seed germination test, we used 3 replicates, each with 72 seeds for each genotype.<br>For primary root length measurement, we used 12 seedlings for each genotype.
Data exclusions	No data were excluded.
Replication	Experimental findings were reliably reproduced as reported as mean +/- sd values of independent replicates. All attempts at replication were successful.
Randomization	All samples for analysis were grouped based on the strain type or genotype. Seeds, seedlings and plants were randomly selected and placed in the plant growth chamber or greenhouse.
Blinding	Blinding was not relevant in this study as it does not affect the behavior of strains or plants, as well as data analysis.

Reporting for specific materials, systems and methods

We require information from authors about some types of materials, experimental systems and methods used in many studies. Here, indicate whether each material, system or method listed is relevant to your study. If you are not sure if a list item applies to your research, read the appropriate section before selecting a response.

Materials & experimental systems

n/a	Involved in the study
☒	☐ Antibodies
☐	☒ Eukaryotic cell lines
☒	☐ Palaeontology and archaeology
☒	☐ Animals and other organisms
☒	☐ Clinical data
☒	☐ Dual use research of concern
☐	☒ Plants

Methods

n/a	Involved in the study
☒	☐ ChIP-seq
☒	☐ Flow cytometry
☒	☐ MRI-based neuroimaging

Eukaryotic cell lines

Policy information about cell lines and Sex and Gender in Research

Cell line source(s)	Saccharomyces cerevisiae strain Y00367 (BY4741; MATa; ura3Δ0; leu2Δ0; his3Δ1; YAL039c::kanMX4) and Y04936 (BY4741; MATa; ura3Δ0; leu2Δ0; his3Δ1; YKL087c::kanMX4) were obtained from Euroscarf (http://www.euroscarf.de). Chlamydomonas reinhardtii strain g1 (CC-5415; nit1, mt+) was obtained from Chlamydomonas Resource Center (https://www.chlamycollection.org/). HCS mutants were generated in our lab through IDT Alt-R CRISPR-Cas9 System. Agrobacterium tumefaciens strain AGL1 and GV3101 was obtained from Intact Genomics.
Authentication	These cell lines are in common use by the investigators for many years. They are repeatedly authenticated through specific growth requirements, antibiotic insensitivities and successful results of complementation and transformation experiments. We confirmed the Chlamydomonas reinhardtii HCS mutants by PCR and sequencing, including appropriate controls, with primer information provided in Supplementary Data File 3.
Mycoplasma contamination	Cell lines were not tested for mycoplasma contamination.
Commonly misidentified lines (See ICLAC register)	No commonly misidentified cell lines were used.

Plants

Seed stocks	Arabidopsis thaliana Col-0 and SALK_046872 line seeds were obtained from the Arabidopsis Biological Resource Center (Columbus, OH). pUBQ10:CriHCCS (complementation line) were generated in our lab. Nicotiana benthamiana plants were obtained from the greenhouse at Ludwig Maximilian University of Munich.
Novel plant genotypes	The complementation line was generated by transgenic expression, employing floral dip transformation in Arabidopsis thaliana. A codon optimized CriHCCS CDS sequence (system III) was synthesized (GenScript, USA) and assembled downstream of the Ubiquitin 10 promoter to create the vector pUBQ10::CriHCCS. The constructed plasmids were then transformed into viable heterozygous ccmh plants (selected by genotyping the progeny of SALK_046872 mentioned above) by the floral dip method via Agrobacterium tumefaciens strain GV3101. Homozygous ccmh plants due to complementation were isolated by selecting seeds on plates containing 10 µM glufosinate ammonium (BASTA; for transformant selection) and PCR genotyping. Progenies from independent transformants were verified for ccmh T-DNA homozygosity and CriHCCS presence by PCR genotyping with the primers listed in Supplementary Data File 3.
Authentication	We confirmed the genotype of homozygous lines by PCR-based genotyping with primer details provided Supplementary Data File 3.
